# Acceptability study of a co-designed educational game about dementia for children: The Kids Dementia Game

**DOI:** 10.1371/journal.pone.0320782

**Published:** 2025-05-08

**Authors:** Patrick Stark, Sonya Clarke, Gary Mitchell, Gillian Carter, Stephanie Craig, Christine Brown Wilson

**Affiliations:** School of Nursing and Midwifery, Queen’s University Belfast, Belfast, Northern Ireland, United Kingdom; University of Basel Institute for Biomedical Ethics: Universitat Basel Institut fur Bio- und Medizinethik, SWITZERLAND

## Abstract

**Background:**

Dementia has physical, psychological, social and economic impacts, not only for people living with dementia, but also for their carers, families and wider society. Due to the growing number of people living with dementia, children are increasingly likely to encounter family members living with dementia. The aim of this project was to pilot an educational game which was co-designed with children and people living with dementia with the intention of improving children’s understanding and perception of dementia.

**Research design and methods:**

An acceptability study of the Kids Dementia Game was conducted in three classes in three schools in Northern Ireland. This study investigated acceptability of the game and the feasibility of online data collection using a pre-post test methodology to explore how best to collect evaluation data if the game was to be delivered on a larger scale.

**Results:**

Evaluation of the game with children showed a positive level of acceptability of the game. Children found the game engaging, easy to navigate and fun to play. Feasibility of the data collection method was found to be a barrier to the pre-post test evaluation of the game.

**Discussion and implications:**

These findings suggest that the game shows evidence of promise for improving public perception and understanding of dementia using an early intervention approach with children.

## Introduction

Many people with dementia live active lives with the support of family, friends, and communities. However, there remain concerns about how they, as people living with dementia are perceived. There is some evidence to suggest that misunderstandings exist in the general public about the capabilities of people living with dementia or how they might continue to meaningfully contribute towards society [1] As the population ages, and the risk of developing dementia increases, there is a pressing need to improve public understanding of what dementia is and knowledge of how to proactively support people in the community living with dementia. Promoting this in younger generations by targeting children and young people may be particularly effective in improving these aspects of public health literacy about dementia. The World Health Organisation (WHO) [2] implemented a plan to address the public health response to dementia with the aim of a dementia-inclusive society by 2025 and one of the WHO global targets places a significant focus on dementia-awareness raising activities.

In addition to the global need to improve public understanding of dementia, it is particularly important to support younger people who may be experiencing dementia in their family with limited support due to a lack of understanding and/or negative perceptions [3].This lack of understanding is detrimental to those living with dementia as it can influence their relationships with both family and friends and their own perceptions of what dementia is [4,5]. Furthermore, when a close family member/ friend develops dementia, the difficulties in dealing with the diagnosis can lead to family distress [6]. Family and friends may avoid contacting the person with dementia and this can leave them feeling isolated, lonely, unsupported and have a negative impact on their wellbeing [7,8]. The World Health Organisation estimates that dementia prevalence is increasing at a rate that will result in 75 million people living with dementia by 2030, and 132 million people by 2050 [2]. As a result of this increasing prevalence, younger people are increasingly likely to encounter friends and relatives who have dementia [9] or to become young carers [10] but this can lead to challenges balancing caring responsibilities and developmental needs [6]. Education may facilitate a young person’s understanding of dementia and empower them to engage with a family member/friend with dementia more effectively as was found by the Kids4dementia learning programme in Australia [11,12]. There is, however, a paucity of age-appropriate resources to support children’s understanding of dementia [13]. There is evidence to suggest that young people struggle to communicate with grandparents living with dementia due to a lack of skill and knowledge, and that their parents have to balance multiple roles to try and address those gaps: young adults, interviewed about their interactions with grandparents or great-grandparents living with dementia highlighted that they experienced a lack of knowledge about dementia and maintaining those relationships [14]. A systematic review highlighted how adult children of people living with dementia had to balance multiple roles – caring for their parents whilst having to teach their own children how to communicate with their grandparents with dementia [15]. The development of positive attitudes through education in younger age groups with an age-appropriate digital dementia game has the potential to prevent the development of negative attitudes [16]. Dementia education has been found to increase empathy and positive attitudes towards dementia by preparing children for interacting with people living with dementia [17]. The current lack of education and support for children to improve their understanding of dementia and to support them in maintaining meaningful relationships with family members with dementia could have negative implications at different levels. If children are not supported to understand dementia, this may have negative implications for their relationships with family members. This may also impact the development of dementia-aware communities, as public health education about dementia is not starting early, and negative attitudes may develop before people encounter effective education about dementia. Early intervention about understanding dementia could aim to eliminate misconceptions about people living with dementia and their capabilities, rather than having to undo these misconceptions in later adulthood.

### The intervention: The Kids Dementia Game

Digital technology is ubiquitous with the current generation of children and young people. Digital games are increasingly being used for learning [18]. Serious digital games are a digitally delivered intervention, designed for a purpose other than entertainment and with the aim of educating or promoting behaviour change [19,20]. Games for learning are a genre of serious games and have been used effectively in primary school education (ages 4–12) to positively influence knowledge and understanding, behaviour change, perceptual and cognitive, motivational and affective domains [21]. Bespoke digital games designed to promote prosocial skills in children demonstrate an improvement in diverse areas such as social self-efficacy, self-confidence and empathy leading in some instances to a change in behaviour [22]. Our previous research demonstrates that coproduction of a short digital game to improve awareness and understanding of dementia (www.dementiagame.com) significantly enhances knowledge and attitudes of the public towards people living with dementia [23,24]. Following the development of this dementia game, our co-design group (including people living with dementia) suggested it was vital for younger age groups to also understand how to support people living with dementia. We engaged children and a group of people living with dementia with experience of grandchildren to codesign a digital game to support children’s understanding about dementia.

This project adapted the codesign process used in the development of the original Dementia Game [23] to include children and people living with dementia in an intergenerational approach. This approach followed three iterative phases. Virtual workshops were undertaken with people with dementia (n = 10) with subsequent face to face workshops undertaken with children from three primary schools in Northern Ireland (one class per school). These schools would later participate in the acceptability testing of the game reported in this present study. In the first co-design phase, the workshops focussed on what information might be most helpful to children with understanding dementia and what children can do to help people living with dementia. In the second phase, workshops focussed on what the most important priorities were in terms of the initial ideas generated in the first phase about key information about dementia and how children might support people living with dementia. In the third phase, an intergenerational workshop was hosted online to reach consensus about the final content of the game. The process was iterative ensuring that at each stage of the game development, both the voice of people living with dementia and the voice of children were central to the design process.

The digital Kids Dementia Game (https://kids.dementiagame.com) is set in a school where children choose child characters to find out about their experience of engaging with a relative living with dementia. The scenarios are in a moving comic book style, enabling the player to move through the game at their own pace. The scenarios are interspersed with questions and answers alongside gameplay elements designed to improve engagement and enjoyment. Engagement and enjoyment of pro-social learning interventions has been found to be significantly predictive of intervention effectiveness [25]. Points are gained through each activity providing a level of competition either with others or themselves. A final score is provided alongside a bronze, silver or gold digital ‘award’. Playing all scenarios takes approximately 15–20 minutes.

## Methodology

This study piloted delivery of the Kids Dementia Game using an acceptability study design, i.e., testing the intervention in the intended setting while also investigating the feasibility of collecting data on effectiveness [26].

Two research questions are addressed in this paper:

RQ1) What is the feasibility of digitally administering an age appropriate questionnaire about dementia before and after playing the game?
RQ2) What is the acceptability of the game to children?

### Ethics statement

Ethical approval was granted from the Faculty Research Ethics Committee of the Faculty of Medicine, Health and Life Sciences of Queen’s University Belfast (approval number: MHLS 21_66) for the evaluation of the Kids Dementia Game.

Written consent was obtained from child participants’ parents/guardians. Assent was obtained from child participants.

### Recruitment

We recruited three classes of children from three schools in Northern Ireland (Demographic characteristics for the sample is shown in Table 1). All schools were in urban areas. The initial demographic survey was completed by n = 86 participants. These schools were recruited after their previous collaboration with the research team during the co-design process of the Kids Dementia Game. The children were recruited from Year 6 classes which have typical age ranges of 9–10 years, however the self-reported ages range from 7 to 12, reflecting possible inaccuracy in this demographic data. The children who participated in this acceptability study, therefore, had previous experience of engaging with people living with dementia. School head teachers acted as gatekeepers and provided potential participant children’s parents with a participant information sheet and form to provide their parental consent. The children (via the parent) were provided with an age-appropriate information sheet and a child assent form following best practice guidance [27]. It was imperative the children were viewed as rights holders, and their views valued [28].

**Table 1:** Demographic information for initial sample of children.

		Count	%
Ethnicity	Any other ethnic group	6	6.98%
	Black Other	1	1.16%
	Chinese	4	4.65%
	Irish Traveller	2	2.33%
	White	73	84.88%
Gender	Boy	27	31.40%
	Girl	49	56.98%
	Prefer not to say	10	11.63%
Age	7	7	5.89%
	8	2	1.92%
	9	11	11.90%
	10	61	73.32%
	11	2	2.64%
	12	3	4.33%

### Data collection

Children from each school were invited to play the game in a computer room in their school. Each child played the game once. A member of the research team (SC) and five pre-registration children’s nursing students supported the children whilst playing the game. Data collection was carried out between 1/5/2022 and 31/10/2022.

### Measures

#### Kids insights into dementia questionnaire.

The participants were directed to complete the ‘Kids Insights into Dementia Questionnaire’ (KIDS) [12] before and after playing the Kids Dementia Game. The KIDS questionnaire was developed and tested with children in primary schools in Australia. KIDS consists of 14 items on a five point Likert scale related to knowledge, attitudes and behavioural intention towards people living with dementia and found to have good concurrent validity and good internal consistency (McDonald’s Omega = .83) [11]. Items were scored on a scale of 1–5, with higher scores reflecting more positive attitudes towards dementia, with possible minimum and maximum scores being 14 and 70 respectively. The pre and post surveys were built into the game for the purposes of the present study. The KIDS questionnaire had previously been designed to be completed using a pen and paper format, but for cost-effectiveness and potential for scaling, the present study piloted the digital administration of the KIDS questionnaire. This provided the opportunity of investigating feasibility of this data collection method at a low-stakes programme development stage, ahead of future efficacy evaluation or scaling-up. This data was used to answer RQ1 (feasibility of data collection).

#### Acceptability of the game: System usability scale.

A set of 10 items based on engagement, enjoyment, ease-of-use, necessary support and likelihood of wanting to play the game again were administered after playing the game (providing data to answer RQ2). These items were adapted from a modified ‘System Usability Scale’ (SUS) [29,30]. SUS is a 10-point scale found to be effective in substantiating the effectiveness and efficiency of a system from the users’ perspective [31]. The adaptation involved including the phrase ‘the game’ in the items of the SUS. This questionnaire was presented digitally to the participants after playing the game and was built into the game web page.

### Data analysis

Quantitative data for the KIDS questionnaire responses before and after playing the game (pre-test and post-test) were matched using a unique user ID and analysed in IBM SPSS version 27. These data were analysed using descriptive statistics and paired sample t-tests. Data from the 10-item adapted SUS were analysed descriptively for proportions of positive and negative responses to each of the ten items. The demographic survey was completed by n = 86 participants, but smaller numbers completed the other quantitative measures: n = 21 completed both pre and post KIDS questionnaire and n = 38 submitted SUS acceptability data and n = 10 of these had missing items. All data collection measures were accessed via the digital game, and there was significant variability in the participants successfully completing these measures, reflected in the different numbers of participants for the demographic, KIDS questionnaires and SUS questionnaire.

## Findings

1) **What is the feasibility of administering the ‘Kids Insights into Dementia (KIDS) Questionnaire’ digitally?**

A sample of n = 27 participants completed the pre-test and n = 26 participants completed the post-test survey and descriptive statistics are shown below in Table 2. Within these participants, n = 21 completed both pre-test and post-test and were able to be included in the paired comparisons. Those who could not be matched completed only a pre-test or a post-test. should be noted that systematically missing data meant that the final item, item 14, could not be analysed, and the below means are for the remaining 13 items with a maximum possible score of 65 rather than 70. Data for the final item was not entered by the majority of participants, due to a technical issue of the online survey being closed early before full completion.

**Table 2:** Mean pre-test and post-test scores for KIDS questionnaire.

	N	Minimum	Maximum	Mean	Standard deviation
Pre-test KIDS	27	41	58	51.89	4.21
Post-test KIDS	26	39	63	53.00	6.52

Overall mean scores remained moderately high from pre-test (M = 51.89, SD = 4.21) to post-test (M = 53.00, SD = 6.52). There was no significant difference between pre-test and post-test scores, as indicated by the paired samples t-test for the n = 21 participants who completed both pre-test and post-test and could be included in this analysis (t[20]=.833, p = .415).

1] **What is the acceptability of the game to children?**

A sample size of n = 38 submitted acceptability scale data and n = 10 had missing item responses. Responses to all positively worded items (i.e., agreement = positive rating of the game) and negatively worded items (i.e., disagreement = positive rating of the game) are shown in Tables 3 and 4. Overall, it can be seen that responses to acceptability of the game were very positive for most items. In terms of enjoyment and engagement with the game, over 90% of participants agreed or strongly agreed that they would use this game a lot. Over 85% felt there was good flow to the game. In terms of ease of use: there were considerably more neutral and negative answers to the items “I found it difficult to follow what I had to do in the game”, “I needed to learn a lot of things before I could get going with the game” and “I think that I would need the support of an adult to use this game” than for other items, although these were still in the minority vs positive responses. This suggests that difficulty and need for support are areas to address in future iterations of the game, albeit for a minority of children. Overall, Tables 3 and 4 show a positive level of engagement with the game in terms of enjoying it, wanting to play it a lot and most participants reported that they did not need additional support and did not struggle with difficulty.

**Table 3:** Acceptability of the Kids Dementia Game (positively worded items).

	Disagree a lot		Disagree		Unsure		Agree		Agree a lot		Total Row N
	Count	Row N %	Count	Row N %	Count	Row N %	Count	Row N %	Count	Row N %	
I think that I would like to use this game a lot.	0	0.0%	0	0.0%	1	2.9%	10	28.6%	24	68.6%	35
I thought the game was easy to use.	0	0.0%	2	5.7%	4	11.4%	12	34.3%	17	48.6%	35
I think most children would learn to use this game very quickly.	0	0.0%	0	0.0%	2	5.6%	10	27.8%	24	66.7%	36
I felt very confident using the game.	0	0.0%	2	5.9%	5	14.7%	12	35.3%	15	44.1%	34
There was a good flow to the game.	0	0.0%	0	0.0%	4	11.1%	13	36.1%	19	52.8%	36

**Table 4:** Acceptability of the Kids Dementia Game (negatively worded items).

	Agree a lot		Agree		Unsure		Disagree		Disagree a lot		Total Row N
	Count	Row N %	Count	Row N %	Count	Row N %	Count	Row N %	Count	Row N %	
I found the game very complicated.	3	8.6%	6	17.1%	1	2.9%	13	37.1%	12	34.3%	35
I think that I would need the support of an adult to use this game.	4	11.1%	3	8.3%	10	27.8%	14	38.9%	5	13.9%	36
I found the game hard to use.	3	8.3%	0	0.0%	3	8.3%	8	22.2%	22	61.1%	36
I needed to learn a lot of things before I could get going with this game.	6	16.2%	2	5.4%	10	27.0%	13	35.1%	6	16.2%	37
I found it difficult to follow what I had to do in the game.	1	2.9%	4	11.4%	6	17.1%	13	37.1%	11	31.4%	35

## Discussion

Our study, focused acceptability testing of the co-designed Kids Dementia Game, aligns with previous research emphasizing the potential of serious digital games in fostering positive attitudes and knowledge about dementia across different age groups [23,32]. The game, co-designed with insights from people living with dementia, allowed us to directly address misconceptions prevalent among children, consistent with the principles of gamification in a pedagogical context [33]. This approach provided a credible alternative to traditional public health teaching methods in child populations, reinforcing the value of lived experiences in enhancing the educational impact of serious games [1,19].

Our study demonstrated the feasibility of recruiting child participants from primary schools, showcasing the convenience and accessibility of serious digital games. Especially when designed for short completion times, these games can encourage multiple plays and outreach to a broader population [19]. The implications of the COVID-19 pandemic further underscore the growing comfort and proficiency of individuals, including children, with online platforms [34]. However, it is crucial to acknowledge a potential limitation associated with this gamified intervention. Unlike previous studies primarily targeting adults, this game’s accessibility for children is contingent on adult gatekeeping [35]. Due to considerations of online safety and potential challenges associated with children navigating the platform independently, adult or teacher support and supervision are likely required for child participants to access the game.

While our study reveals insights, the absence of statistically significant effects indicates the need for cautious interpretation. Further research is essential to validate the effectiveness of the Kids Dementia Game through additional testing, through a larger scale evaluation. Future iterations may consider tailoring the game for different young audiences, including child carers and teenagers as have been used in other public health initiatives [36].

This study found that the digital version of the KIDS questionnaire that was piloted was not feasible for use with this age range of children (mean age 9 years 8 months). The embedding of multiple surveys in the game was challenging for children to complete and resulted in high proportion of missing data The process of completing a demographic survey, pre-test, playing the game then two further surveys was not a feasible task for children this age to complete unassisted. Avoiding this missing data may have required one to one support from teachers or researchers which would reduce the feasibility of collecting data on a large scale in a suitable time frame. In future, a data collection approach aligned with the original pen and paper methods used by Baker et al. [12] is likely to mitigate this issue and result in a more robust dataset for analysis of efficacy.

Comparing our study to two adult-focused studies on serious dementia games, we observe consistent themes. In both adult studies, game-based interventions led to significant increases in dementia knowledge, indicating the potential of digital games in education [23,32]. The co-design aspect, involving the ‘lived experience’ of dementia, emerges as a common strength in enhancing the meaningfulness of serious games in both adult and child populations [32].

### Limitations

Acceptability studies, such as this present study, have inherent limitations and should be treated with cautious interpretation, especially when data collection for measures of intervention efficacy have been piloted. Although this study collected pre-test and post-test data on changes to attitudes about Dementia and piloted an approach to testing for efficacy using paired comparisons, the study was not powered to detect such an effect and so this result should be interpreted with caution. Furthermore, the finding that the feasibility of data collection was low with the piloted method was associated with significant missing data which further impacted the piloting of the paired comparison approach for measuring intervention efficacy. Thirdly, the sample in this acceptability study was recruited from the schools who had been involved in the co-design process, and this is a source of potential bias. It is possible that participant attitudes towards Dementia and their rating of the acceptability of the game could be different from the wider population due to prior involvement in the co-design process.

## Conclusions

Overall, this study shows that the co-designed Kids Dementia Game was acceptable to children as a medium for delivering education about dementia in a primary school setting. These findings, alongside the investigation of data collection feasibility, will be used to inform a larger study of this intervention, such as a feasibility study to investigate the scalability of the intervention with a wider sample. While our study focuses on the ‘serious’ aspect of the game, future evaluations should also consider the ‘entertaining’ element and the overall usability of the game, as programme engagement has been found to be a significant predictor of learning outcomes [25,37]. Addressing the varying needs and attitudes of learners is crucial for developing pedagogically effective serious games [38]. A holistic approach to evaluation, including considerations of entertainment, usability, and children’s attitudes, will provide a more comprehensive understanding of serious games’ impact on education.

## Supporting information

S1 File KIDS survey data. (SAV)

S2 File SUS acceptability data. (SAV)
